# An Online Family Literacy and Wellness Program for Latino Dual Language Learners: Pilot Randomized Waitlist Controlled Trial

**DOI:** 10.2196/60764

**Published:** 2025-06-19

**Authors:** Kevin D Guerrero, Lucia Lakata, Daniel Lima, Caroline Mendoza, Nila Uthirasamy, Lesley M Morrow, Silvia Perez-Cortes, Maria Pellerano, Alicja Bator, Pamela Ohman Strickland, Benjamin F Crabtree, Manuel E Jimenez

**Affiliations:** 1 Rutgers Robert Wood Johnson Medical School, New Brunswick, NJ, United States; 2 The Boggs Center on Disability and Human Development, Department of Pediatrics, Rutgers Robert Wood Johnson Medical School, Liberty Plaza – 335 George Street, 3rd Floor, New Brunswick, NJ, 08901, United States, 1 732-235-9300; 3 Graduate School of Education, Rutgers University, New Brunswick, NJ, United States; 4 Camden College of Arts and Sciences, Rutgers University, Camden, NJ, United States; 5 Department of Family Medicine and Community Health, Rutgers Robert Wood Johnson Medical School, New Brunswick, NJ, United States; 6 Rutgers School of Public Health, Piscataway, NJ, United States

**Keywords:** pediatrics, children, family, English as a second language, ESL, child development, development, wellness, health equity, dual language learner, literacy, language, Latinos, Spanish, Hispanic, randomized, controlled trials

## Abstract

**Background:**

Early childhood interventions can simultaneously promote positive health and early language experiences, but implementation and health equity often receive insufficient attention during the development process.

**Objective:**

We apply a health equity lens to refine and pilot-test a family literacy and wellness program designed for Latino dual language learners (DLLs) entering kindergarten and their caregivers.

**Methods:**

In collaboration with a parent and community advisory board, we refined an 8-week family literacy and wellness program and conducted a pilot randomized controlled trial (RCT) with a waitlist control. The program, specifically designed by our interprofessional team for Latino DLLs, uses health topics (ie, nutrition, physical activity, sleep, and social-emotional development) to (1) introduce foundational language and literacy skills to children; (2) empower families to engage in health and home literacy activities using a strengths-based approach; and (3) encourage maintenance of families’ home language. We assessed reach by collecting sociodemographic information; attendance and acceptability using a parent survey; and preliminary effects on home literacy activities through a validated parent-report instrument (StimQ_2_ quantity, quality, content, and concepts subdomains) and on child literacy skills using investigator-developed assessments. We analyzed quantitative data using descriptive statistics and regression analyses.

**Results:**

Parents and community advisors informed the program content. A total of 32 parent-child dyads were enrolled in the pilot RCT. All parents identified as Latino, and half had not completed high school, indicating that we reached the intended audience. Parents rated the program as highly acceptable, and 23 (72%) participants attended at least half of the sessions. After participation, group 1 had higher StimQ_2_ quality scores (effect size 0.99, *P*=.02) and higher quantity scores (effect size 1.01, *P*=.04) compared with group 2.

**Conclusions:**

Similar interprofessional collaborations may be a promising strategy to promote equity in early language experiences for Latino DLLs and their families.

## Introduction

Education is critically important for health and well-being, yet this social driver of health has received insufficient attention in health care settings [1]. Kindergarten performance is a strong predictor of health risk behaviors and indicators of well-being, such as college education [2-4]. As a result, existing inequities in kindergarten readiness [5,6] pose a threat to long-term health and well-being at both individual and population levels.

The US Department of Health and Human Services and the US Department of Education define dual language learners (DLLs) as children with a home language other than English who are learning 2 or more languages simultaneously or learning a second language while still developing their first language [7]. Latino DLLs are a rapidly growing segment of the population who face discrimination and unequal opportunities, predisposing them to poor educational, occupational, and health outcomes [8,9]. Sustainable interventions to support this population are therefore urgently needed.

Pediatric clinicians are in a unique position to implement early childhood interventions that promote optimal school readiness, given their near-universal access to young children, frequent contact with families, and opportunities to build and leverage strong parent-clinician relationships [10-12]. Several programs are designed to be embedded within early childhood settings to promote early language development among DLLs [13,14]. However, early childhood interventions can simultaneously promote both physical and academic school readiness. Interventions focused on family literacy offer a clear example. Family literacy can be defined as the way families use literacy in their homes and communities, including during typical routines [15-17]. Families already engage in a wide range of literacy activities that are present in many health-related routines (eg, reading food labels). Embedding these practices into family literacy programs may provide a more ecologically valid intervention—one rooted in existing rather than entirely new practices and one that embraces cultural diversity. Few early childhood interventions take advantage of these opportunities. One example is a program that uses culturally sensitive, typical family food routines to support children’s language and literacy skills, such as vocabulary, decoding, and writing [18]. Programs can adopt this approach to engage families and build cross-sector partnerships between pediatric professionals and educators—partnerships that find synergy in their complementary expertise. Despite their potential to promote equity through family engagement, such cross-sector partnerships remain rare.

Implementation often receives insufficient attention early in the development of health promotion interventions. This is critical, as a limited understanding of community context and partner priorities can potentially diminish—or even eliminate—an intervention’s impact [19]. Baumann and Cabassa [19] identified key elements that can support the integration of an equity lens early in implementation research, including a focus on reach from the outset and the intentional design of interventions with historically marginalized populations in mind. Partnered approaches from the earliest stages of intervention design and refinement can help promote health equity, yet they remain rare. To our knowledge, few early childhood interventions have leveraged this strategy.

To address these gaps, we applied implementation science with a health equity lens to refine and pilot-test an online family literacy and wellness program designed for Latino DLLs entering kindergarten and their families. Using community-engaged research strategies, we partnered with parents to refine the program and subsequently conducted a pilot randomized controlled trial (RCT). Consistent with the approach proposed by Baumann and Cabassa [19], we intentionally focused on intervention reach, design, and equity-relevant implementation outcomes. These insights can inform similar cross-sector education-health care partnerships that aim to promote equity for Latino DLLs and their families.

## Methods

### Study Design and Registration

We conducted a pilot study using an RCT with a waitlist control design.

### Ethical Considerations

The Rutgers Health Institutional Review Board approved this study (approval number Pro2021001575). All participants provided informed consent. Data were deidentified. Participants received a US $25 retail gift card for each study visit completed. The study was registered before enrollment of the first participant at ClinicalTrials.gov (NCT05339464). We followed CONSORT (Consolidated Standards of Reporting Trials) guidelines for reporting clinical trials [20].

### Study Population and Setting

We recruited Latino DLLs (aged 4‐6 years) and their parents from Eric B. Chandler Health Center, a local Federally Qualified Health Center, and the surrounding greater New Brunswick area. Eric B. Chandler Health Center primarily serves Latino individuals from underresourced communities. Clinicians at the center referred potentially eligible participants to the study team. We also advertised the study with our local community partners using recruitment flyers and word of mouth. Eligibility criteria included primary caregivers aged ≥18 years (referred to as parents for the remainder of this article) of children entering kindergarten who identified as Latino, used Spanish at home, owned a cellphone, and were willing to receive SMS text messages and be randomized. We excluded children with multiple anomalies or genetic disorders, as well as those with previously identified developmental delays.

### Program Refinement

We partnered with our parent advisory board through a series of community engagement studios that helped refine the program throughout the project [21]. The parent advisory board consists of parents who participated in past iterations of the family literacy and wellness program. All parents identify as Latino and prefer Spanish as their primary language for communication. Their children are in kindergarten, first grade, or second grade, providing a range of scholastic experiences and perspectives on their children’s needs. We conducted 7 community engagement studios with our parent advisory board from January 6, 2021, to January 30, 2023. We adapted the community engagement studio concept from the approach developed by the Meharry-Vanderbilt Community Engaged Research Core [21] to facilitate meaningful participation and engagement. Each studio focused on a specific aspect of the program. Bilingual team members presented relevant information about the program, and a bilingual research coordinator then facilitated dialogue to elicit parents’ feedback on the content and procedures. Through this process, parent advisors provided substantive input on the program’s mission, intervention content, and logistics. The project also includes a community advisory board composed of local community leaders, including educators, a pediatrician, a librarian, and a community health expert, that meets regularly to advise the team on outreach and engagement strategies, as well as program content.

### Study Conditions

All parent-child dyads participated in the family literacy and wellness program, Ready and Healthy for Kindergarten. Parent-child dyads were enrolled and randomized 1:1 to either the first group (June-July) or the second group (July-August). The randomization schedule was computer generated by the study biostatistician (POS). Allocation concealment was maintained using the REDCap (Research Electronic Data Capture; Vanderbilt University) randomization module. Participants in both groups received a book bag with school supplies, an activity kit, and program books at enrollment. The program was specifically designed by our interdisciplinary team (ie, education, linguistics, and pediatrics) for Latino DLLs, using health topics (ie, nutrition, physical activity, sleep, and social-emotional development) to (1) introduce and reinforce foundational language and literacy skills to children, (2) empower families to engage in health and home literacy activities using a strengths-based approach, and (3) encourage the maintenance of families’ home language and cultural traditions [22,23]. The program is designed to be strengths-based by building on families existing routines and inviting caregivers to incorporate new ones that support their goals and the objectives listed above. For example, during the nutrition topic, teachers may highlight how following a family recipe during meal preparation, whether written or recalled from memory, offers embedded opportunities for sequencing tasks, storytelling, and interaction. During the physical activity topic, teachers may point out how everyday simple family activities, such as walking to the bus stop, can include literacy-rich moments like reading street signs. Each session follows a predictable structure that draws out families’ existing routines. Teachers use language flexibly and authentically, in alignment with the families’ preferences, to tacitly normalize bilingualism and position home language maintenance as the standard, thereby empowering participation. In addition, the social-emotional unit includes a dedicated session focused on cultural pride, honoring one’s heritage, and highlighting the advantages of multilingualism, explicitly encouraging the maintenance of the home language.

We first offered the program in person at Eric B. Chandler Health Center in 2019 [22]. This initial iteration established the program structure, which included (1) 8 parent-child workshops; (2) take-home kits, including a book bag with school supplies, activity kit, and program books, to support at-home extension activities that reinforced session content; and (3) reminder SMS text messages that reinforced in-class content [22]. In 2020, in response to the COVID-19 pandemic, the workshops shifted to an online format using videoconferencing software [23]. We retained the virtual format for the 8 parent-child workshops, which we then refined and pilot-tested in this study.

### Data Collection

Trained bilingual research assistants collected data online via secure videoconference software at 3 time points: enrollment (study visit 1); approximately 2 months after enrollment, after group 1 completed the program and before group 2 began (study visit 2); and approximately 4 months after enrollment, after group 2 completed the program (study visit 3). Recruitment began in March 2024, the first study visit occurred on May 9, 2022, and the last study visit took place on September 25, 2022. In this paper, we focus on between-group differences in parent literacy and language activities and child outcomes at study visit 2, which captures the period after group 1 completed the program and before group 2 began.

### Implementation Outcomes

#### Reach

As noted by Baumann and Cabassa [19], equity research requires attention to reach, that is, who is included in and participating in research. To assess whether the program reached its intended audience, we examined demographic characteristics of parent-child dyads, including ethnicity, parent education, and self-reported English proficiency.

#### Attendance

To understand the extent to which parents and children would use the program, we examined session attendance. A study team member documented attendance at each session.

#### Acceptability

Acceptability can be defined as users’ perception of the extent to which a treatment or service is satisfactory [24]. In this study, we assessed acceptability using the Acceptability of Intervention Measure (AIM) [25], a validated 4-item survey that evaluates participants’ perceptions of program acceptability.

### Parenting and Child Outcomes

#### Parenting Outcomes

The StimQ_2_ is a validated parent-report measure of the home cognitive environment, available in both English and Spanish [26]. In the validation sample, 93% of caregivers identified as Latino, 76% completed the surveys in Spanish, and 91% were classified as having low socioeconomic status based on the Hollingshead Four Factor Index. We used the StimQ_2_ READ scale to explore the program’s effects on parent-home literacy activities, focusing on subdomains that assess quantity, quality, content, and concepts. We used the StimQ_2_ Parent Verbal Responsivity (PVR) scale to examine verbal responsivity during parent-child interactions. Both the READ and PVR subscales demonstrate good reliability (Cronbach α=0.753 and 0.790, respectively) and strong validity, as indicated by correlations with assessments of child language, cognitive ability, and social-emotional skills.

#### Child Outcomes

We used investigator-developed assessments to explore the program’s effect on child outcomes. These assessments were administered by a bilingual research assistant in the child’s preferred language. Here, we focus on tests of children’s development in letter identification, letter sound identification, thematic vocabulary identification, and book awareness. To assess book awareness, children were asked to identify different print concepts (ie, front and back cover, title page) using a book of their choice. The examination of letter and sound identification involved an untimed task in which children were asked to identify 10 different letters (presented in upper and lower case) and to produce their corresponding sounds. Vocabulary identification, focusing on words discussed during the workshops, such as healthy habits, foods, and family routines, was assessed using a picture-based vocabulary recognition task.

#### Sample Size

In determining our sample size, we considered the main objective of this pilot study, to examine equity-relevant implementation outcomes such as reach, engagement, and acceptability, and to explore the program’s preliminary effects on parenting and child outcomes in an exploratory manner. We also followed best practice recommendations [27,28]. Based on these factors, our goal was to randomize at least 24 participants (12 per arm). The recruitment response exceeded our expectations, and we enrolled more participants to avoid turning interested families away.

### Data Analysis

We first calculated means and SDs or percentages for continuous or categorical variables to examine reach using sociodemographic data, engagement using attendance logs, and acceptability using AIM survey responses. Participants were analyzed according to the group to which they were originally randomized. Participants who discontinued or were lost to follow-up were excluded from analyses. We used regression models to explore between-group differences in home literacy activities measured by the StimQ_2_ READ scale total score and its subdomains, quantity, quality, diversity of concepts, and diversity of content, as well as responsive verbal interactions assessed by the StimQ_2_ PVR. In addition, child literacy skills were evaluated using investigator-developed assessments focused on letter identification, letter-sound knowledge, vocabulary, and book awareness. We adjusted for the following important a priori–identified covariates*:* baseline scores, child age, child language, and parent education. To enable comparison across outcomes, we calculated effect size estimates by dividing the treatment coefficient from the regression models by the residual SD of each outcome.

## Results

### Program Refinement

The parent advisory board helped shape the program’s logistics and content. Drawing on their experience with the program, parents provided specific feedback that guided decisions about session length, program duration, and optimal dates and times for the online sessions. Synthesizing this feedback, we kept sessions under 1 hour, offered multiple evening options on weekdays, and scheduled Saturday sessions in the late morning or around noon. Parents also provided guidance and feedback on recruitment strategies and materials, which helped enhance reach. During meetings, they also offered input that informed adjustments to instructional pace, use of materials, and routines for parent-child interactions. Key considerations were whether the materials were easy to read and use at home. For example, children enjoyed opportunities to draw pictures starting with the session’s focus letter, and parents shared that their children liked cutting out cards to practice letter sounds. As a result, we adapted the session content and at-home activity kits to include more opportunities for these activities. Families found the at-home extension activities enjoyable and identified them as a strength. Parents also reported that the school supplies sent home in the backpack were useful for their home learning environment.

### Pilot Study

**Figure 1.**
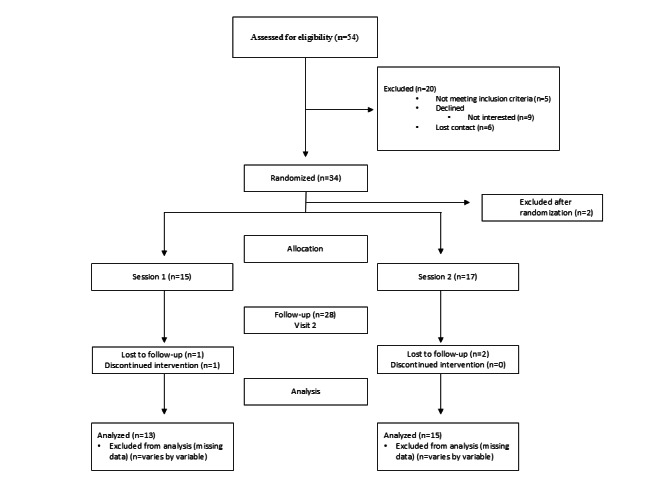
CONSORT (Consolidated Standards of Reporting Trials) flowchart.

A total of 54 parent-child dyads initially expressed interest in participating; however, 5 of these dyads did not meet the inclusion criteria, 6 were lost to contact, and 9 declined participation. The most common reason for declining was lack of interest. Ultimately, we randomized 34 parent-child dyads (Figure 1). Two parent-child dyads were later excluded for not meeting eligibility criteria, resulting in a final enrolled sample of 32 dyads. Among these, 3 parent-child dyad groups were lost to follow-up, and 1 dyad discontinued participation, leaving 13 dyads in group 1, and 15 dyads in group 2.

### Reach

Demographic characteristics are summarized in Table 1. All participants identified as Latino (N=32), with 16 of 32 (50%) reporting Mexican origin; 29 out of 32 (91%) reported speaking English less than very well, and 17 (53%) had not completed high school. At enrollment, children were on average 4.5 years old, and 20 (63%) were identified as native-like Spanish speakers. These characteristics suggest that the program reached the intended audience.

**Table 1.** Demographic information of study participants at enrollment.

	Total (n=32)	Group 1 (n=15)	Group 2 (n=17)
Child age (years), mean (SD)	4.5 (0.5)	4.6 (0.5)	4.5 (0.5)
Child sex, n (%)			
Male	17 (53)	7 (47)	10 (59)
Female	15 (47)	8 (53)	7 (41)
Child’s ability to speak Spanish, n (%)			
Speaks like a native speaker	20 (63)	7 (47)	13 (76)
Almost like a native speaker	6 (19)	4 (27)	2 (12)
Difficulty speaking it	5 (16)	3 (20)	2 (12)
Barely speaks it	1 (3)	1 (7)	0 (0)
Child’s ability to understand Spanish, n (%)			
Understands it like a native speaker	22 (69)	9 (60)	13 (76)
Understands it for the most part	5 (16)	3 (20)	2 (12)
Difficulty understanding it	3 (9)	2 (13)	1 (6)
Barely understands it	2 (6)	1 (7)	1 (6)
Preschool attendance, n (%)			
Beginning at age 3	19 (59)	10 (67)	9 (53)
Beginning at age 4	9 (28)	4 (27)	5 (29)
Never attended	4 (13)	1 (7)	3 (18)
Parent’s age (years), n (%)			
21-25	5 (16)	3 (20)	2 (12)
26-30	5 (16)	2 (13)	3 (18)
31-40	22 (69)	10 (67)	12 (71)
Parent’s country of birth, n (%)			
Mexico	16 (50)	7 (47)	9 (53)
Honduras	5 (16)	3 (20)	2 (12)
Other	9 (28)	3 (20)	6 (35)
United States	2 (6)	2 (13)	0 (0)
Parent-reported English proficiency^a^, n (%)			
Very well	3 (9)	2 (13)	1 (6)
Well	5 (16)	2 (13)	3 (18)
Not well	12 (38)	7 (47)	5 (29)
Not at all	12 (38)	4 (27)	8 (47)
Parent’s highest level of education, n (%)			
Less than eighth grade	7 (22)	3 (20)	4 (24)
Ninth to twelfth grade (no diploma)	10 (31)	2 (13)	8 (47)
High school diploma or greater	15 (47)	10 (67)	5 (29)

a Parents’ response to “How well do you speak English?”

### Attendance

Program attendance is summarized in Table 2. Seventy-two percent of parents (23/32) attended at least half of the sessions. Timing appeared to influence attendance, with lower participation observed in the second half of the summer. Some families reported traveling internationally or taking on additional seasonal work during this period, which may have impacted their ability to attend.

**Table 2.** Number of workshops attended by participants during the family literacy and wellness program (n=32).

Number of sessions attended	Number of participants
0	4
1	1
2	2
3	2
4	3
5	2
6	5
7	5
8	8

### Acceptability

On average, parents agreed that the program met their approval, it was appealing, they liked the program, and they welcomed what they learned (Table 3).

**Table 3.** Acceptability of intervention survey among the study population (n=25 parents).

AIM^a^ question	AIM score, mean (SD); range^b^
The program met my approval.	3.5 (0.5); 3-4
The program was appealing to me.	3.5 (0.5); 3-4
I liked the program.	3.5 (0.5); 3-4
I welcome what I learned in the program.	3.5 (0.5); 3-4

a AIM: Acceptability of Intervention Measure.

b 0=completely disagree, 1=disagree, 2=neither agree nor disagree, 3=agree, and 4=completely agree.

### Parenting Outcomes

Results of the StimQ_2_ READ and PVR scales are presented in Table 4. Although not statistically significant, there was a moderate difference on the total StimQ_2_ READ scale between group 1 (ie, those who completed the program) and group 2 (ie, those who had not yet begun; Cohen *d*=0.55; *P*=.23). However, there were large and statistically significant differences between the groups on the quantity and quality subdomains (Cohen *d*=1.01; *P*=.04 and Cohen *d*=0.99; *P*=.02). By contrast, the between-group difference on the PVR scale was small and not statistically significant (*P*=.75).

**Table 4.** Effects of Ready and Healthy for Kindergarten participation (group 1) compared with waitlist control (group 2) on the Home Literacy Environment and Parent Verbal Responsivity scale scores.^a^

Measures	Group 1, effect size estimate (95% CI)	*P* value
StimQ_2_ READ scale^b^		
Total score	0.55 (−0.44 to 1.10)	.23
Subdimensions		
Book reading quantity	1.01 (0.05 to 1.52)	.04
Book reading concepts	−0.20 (−1.41 to 0.52)	.66
Book reading content	0.02 (−1.09 to 0.68)	.95
Book reading quality	0.99 (0.18 to 1.41)	.02
StimQ_2_ Parent Verbal Responsivity Scale^b^	0.14 (−0.92 to 0.76)	.75

a Linear regression analyses were conducted, adjusting for baseline scores, child age, child language, and parent education. Effect size estimates were calculated by dividing the treatment coefficient from the regression models by the residual SD of each outcome.

b Subscale of the StimQ_2_-Preschool, a parent-report questionnaire that assesses the cognitive home environment for children aged 36-72 months.

### Child Outcomes

Child outcomes are summarized in Table 5. Between-group differences in letter identification, letter-sound knowledge, and vocabulary were either minimal or in an unexpected direction. However, a moderate-to-large effect, though not statistically significant, was observed for book awareness (Cohen *d*=0.82; *P*=.11).

**Table 5.** Effects of Ready and Healthy for Kindergarten participation (group 1) compared with waitlist control (group 2) on child skills.^a^

	Group 1, effect size estimate (95% CI)	*P* value
Letters identified	0.07 (−1.07 to 0.71)	.88
Letters sounds identified	−0.03 (−1.18 to 0.64)	.95
Vocabulary words identified	−1.68 (−5.84 to 0.21)	.10
Book awareness	0.82 (−0.23 to 1.35)	.11

a Linear regression analyses were conducted, adjusting for baseline scores, child age, child language, and parent education. Effect size estimates were calculated by dividing the treatment coefficient from the regression models by the residual SD of each outcome.

## Discussion

### Principal Findings

In this study, we refined an online family literacy and wellness program using parent feedback. During pilot testing, we found that the program effectively reached and engaged Latino DLLs and their families. We also observed promising improvements in parent home literacy activities after participation. The intentional involvement of families and community partners throughout the research process, along with a deliberate focus on reach and other early implementation outcomes, provided valuable insights into applying a health equity lens to early childhood interventions. These findings offer guidance for interdisciplinary teams aiming to build cross-sector collaborations and move beyond siloed efforts in the pursuit of equity.

The US Centers for Disease Control and Prevention defines health equity as “the state in which everyone has a fair and just opportunity to attain their highest level of health” [29]. The Robert Wood Johnson Foundation elaborates that achieving health equity “requires removing obstacles to health such as poverty, discrimination, and their consequences, including powerlessness and lack of access to good jobs with fair pay, quality education and housing, safe environments, and health care” [30]. Such obstacles drive health inequities, and health care organizations have developed programs in response to growing evidence that social needs impact health [31]. However, health care interventions have largely missed the opportunity to address educational achievement, an important social driver of health that underlies both social and health outcomes [32]. As a cross-sector partnership between health and education, Ready and Healthy for Kindergarten aims to promote health equity by coaching families on how to implement healthy routines early, while supporting children’s development of literacy and numeracy skills that foster health literacy and wellness throughout their life course.

All caregivers in our sample identified as Latino, and half had not completed high school, indicating that the program successfully reached the intended audience. The family literacy and wellness program was designed to address the specific needs of this population, with careful consideration of their linguistic and ethnic backgrounds. Central to this approach, our program is bilingual, supporting children’s language development while honoring and accommodating parents’ linguistic preferences. The focus on families from Latino backgrounds encompassed not only the language of implementation but also the cultural aspects embedded in the program. Meta-analyses support that family literacy programs increase parent literacy activities and improve child emergent literacy [33,34]. However, low participation and engagement among Latino parents from underresourced communities have been major weaknesses of these programs [35]. There are important modifiable program design weaknesses [18]. First, similar programs have been criticized for taking a deficit approach and imposing dominant cultural activities on families without building on their unique strengths [35,36]. Such approaches do not take into account parents’ experiences with activities such as reading, which may be negative [37]. Second, while bilingual programs are emerging, most do not build on families’ heritage language, which is a major issue [38-41]. Third, there is a paucity of studies focused on engagement with Latino families from underresourced communities [18]. By engaging parents as partners throughout the research process and refining the program based on their feedback, we were able to overcome many of these barriers and reach the intended audience with high attendance levels.

Consistent with previous work, we found that the online family literacy and wellness program was well attended and highly acceptable to families [22,23]. This study extends our prior work by demonstrating promising patterns in both the quantity and quality of home literacy activities. Although we did not identify differences in letter identification, letter-sound knowledge, and vocabulary, it is possible that 8 weeks is not sufficient time to detect these differences. Although not statistically significant, the moderate to large effect on book awareness aligns with the enhanced quantity and quality of home literacy activities. One possibility is that by increasing parent literacy activities during the summer before kindergarten, improvements in child outcomes may be observed later. Future studies will need to follow children for a longer period to test this hypothesis—an issue we are currently addressing in our ongoing work.

This work is subject to certain limitations. First, while pervasive inequities in school readiness and wellness provide a strong rationale for focusing on Latino DLLs and their families, our findings may not generalize to all settings or other populations. Future work is needed to tailor program content for participants from different racial and ethnic backgrounds. Second, as this was a pilot study, the primary focus was on examining equity-focused implementation outcomes including reach, attendance, and acceptability. While a sample size of 32 aligns with recommendations for pilot studies [27,28], it is possible that the statistical analyses did not have adequate power to detect differences in parent and child outcomes that may be meaningful. Further, we were unable to examine a threshold for the dose of the intervention necessary to affect outcomes; we are addressing this limitation in our ongoing work. Third, we relied on parent-report measures for home literacy activities and health routines, which may be subject to recall bias and social desirability. Future work should incorporate observational measures to help address this limitation. Fourth, engagement with an intervention goes beyond usage and must capture the relationship between the program and the intended goal of the intervention [42], in this case empowering caregivers to build on their existing strengths and support their children’s physical health and school readiness. While this was beyond the scope of this pilot study, we plan to address it in future work.

### Conclusion

We found that a family literacy and wellness program designed for Latino DLLs reached the intended audience, achieved strong attendance, and showed promising patterns in home literacy activities. While additional work is needed to definitively test the program’s effects and identify optimal implementation strategies, these findings suggest that such interdisciplinary collaborations could be a promising approach to promote equity in children’s early language experiences.

## Supplementary material

10.2196/60764 Checklist 1 CONSORT-EHEALTH checklist.

## Abbreviations

AIM: Acceptability of Intervention Measure
CONSORT: Consolidated Standards of Reporting Trials
DLL: dual language learner
PVR: Parent Verbal Responsivity
RCT: randomized controlled trial
REDCap: Research Electronic Data Capture
